# Psychosocial and occupational predictors of health among Spanish University Employees: The role of job type, contract status, and seniority

**DOI:** 10.1371/journal.pone.0339726

**Published:** 2026-02-02

**Authors:** Ángela Asensio-Martínez, Alejandra Aguilar-Latorre, Barbara Masluk, Sandra León-Herrera, Raquel Sánchez Recio, Bárbara Oliván-Blázquez

**Affiliations:** 1 Institute for Health Research Aragón (IIS Aragón), Zaragoza, Spain; 2 Research Network on Chronicity, Primary Care and Health Promotion (RICAPPS, RD24/0005/0004), Carlos III Health Institute, Madrid, Spain; 3 Department of Psychology and Sociology, Faculty of Social and Labor Sciences, University of Zaragoza, Zaragoza, Spain; 4 Department of Psychology and Sociology, Faculty of Human Sciences and Education of Huesca, University of Zaragoza, Huesca, Spain; 5 Department of Psychology and Sociology, Faculty of Education, University of Zaragoza, Zaragoza, Spain; 6 Research Group on Health Services in Aragon (GRISSA), Department of Preventive Medicine and Public Health, Faculty of Social and Labor Sciences, Universidad de Zaragoza, Zaragoza, Spain; PLoS ONE, UNITED STATES OF AMERICA

## Abstract

The digital transformation of work environments, particularly in academia, has introduced new psychosocial risks, including technostress. This cross-sectional study analyses the relationship between technostress and general health among university employees at the University of Zaragoza, examining the moderating effects of job type, contract status, and seniority. A total of 458 staff members, including academic and research staff and administrative and service staff, completed an online survey assessing general health, technostress, perceived stress, burnout, job satisfaction, resilience, social support, and other occupational variables. The results revealed that perceived stress was the most consistent predictor of emotional symptomatology across all subgroups. Technostress also emerged as a significant predictor, especially among permanent employees and those with longer tenure. Conversely, resilience and job satisfaction functioned as protective factors. Differences in health outcomes were observed depending on employment conditions, highlighting the importance of organizational context. These findings underscore the need for tailored interventions to mitigate psychosocial risks associated with information and communication technologies use and to enhance well-being in academic institutions. This study contributes to the growing evidence base on the health implications of digital work environments in the higher education sector.

## Introduction

In recent years, academic work conditions for university employees have undergone significant transformations, driven by the integration of increasingly sophisticated technological solutions that reshape work dynamics and methodologies within and beyond the classroom. Workplace risk factors are closely linked to both physical and psychosocial safety conditions, including environmental aspects, physical, chemical, and biological agents, as well as psychosocial origins and organizational characteristics [1,2]. Likewise, the labour market and organizations are evolving in a highly volatile environment, characterized by technological innovations that demand new prevention and advisory measures to avoid the risks arising from the technological impact on business effectiveness and the psychosocial needs of workers [3].

In this context, the use of information and communication technologies (ICT) and the adoption of teleworking have increased significantly, especially in the labour and education sectors [4]. This phenomenon has reignited the debate around the right to digital disconnection and the increase in stress associated with these new work modalities, including burnout [5].

The development of ICT has contributed to additional stress, known as technostress, stemming from the need to adapt to new technologies. This concept was defined by Bros, in 1984 [6], as ‘a disease of adaptation caused by an inability to cope with new computer technologies in a healthy way’ [6]. However, it is not ICT itself that produces negative or positive outcomes, but rather the difficulty in managing it healthily, both professionally and personally. Thus, technostress results from a combination of anxiety, information overload, role conflict, and organizational factors [7,8]. The rapid evolution of ICT minimizes the time available for adaptation, thereby increasing levels of technostress [9]. This state is driven by the perceived mismatch between the demands and the resources related to the use of ICT, leading to a high level of unpleasant psychophysiological arousal, increased cardiovascular activity, and elevated levels of stress hormones such as adrenaline and cortisol, along with negative attitudes toward ICT [10,11]. These observations are consistent with the theory of Lazarus and Folkman, who conceptualized stress as the response that occurs when environmental demands exceed the available resources to cope with them [12].

Technostress has consequences for both mental and physical health, as well as work outcomes. It is associated with increased anxiety, depression, and physical health problems such as headaches and sleep disorders [13–16]. In addition, it can reduce job satisfaction, productivity, and performance, while increasing work-life conflicts and intentions to leave the job [14,16–18]. However, it has been observed that factors such as digital competence, resilience, and organizational support can help mitigate these effects [14,19].

Regarding the prevalence of technostress, a study conducted by Affor and Independent Trade Union and Civil Servants’ Association in 2021, which included approximately 800 Spanish workers, showed that technostress affected 72.3% of the active workforce, with the education and healthcare sectors being the most affected [20]. In particular, various recent studies indicate that teachers in Spain experience levels of technostress ranging from moderate to high (Penado Abilleira et al., 2021; Rey-Merchán & López-Arquillos, 2022; Solís et al., 2023). These figures align with previous studies conducted in Europe, which have found similar levels of technostress [21,22].

Previous literature has mainly focused on describing the antecedents and work-related consequences of technostress, but there is a noticeable lack of empirical studies evaluating its relationship with health as a risk factor, especially among employees in general and university staff in particular. This knowledge gap limits the development of appropriate strategies for managing technostress [23,24]. It is necessary to delve deeper into this area, so the main objective of this study is to analyse the levels of technostress among university workers and its relationship with health, as well as psychological and work-related factors.

## Materials and methods

### Design

A cross-sectional, descriptive, quantitative, and retrospective study was conducted using a self-administered online survey during 1 June 2022 and 31 July 2022.

### Participants

This contextual study was conducted with a representative sample of university workers at the University of Zaragoza, aged over 18. The sample size was calculated based on data from the University of Zaragoza, which reported a total workforce of 6,007 employees in 2020: 4,296 in academic and research staff (ARS), and 1,711 in administrative and service staff (ASS). With a 5% margin of error, a 95% confidence level, and a precision of 3%, a minimum sample of 206 individuals was required.

Inclusion criteria were signing the electronic informed consent, proficiency in written and spoken Spanish, and being contracted staff (either teaching and research or administrative and service personnel) at the University of Zaragoza.

### Procedure

A non-probabilistic convenience sampling method was used. The invitation was distributed through internal communication channels of the University of Zaragoza (bulletin board, newsletter, and mailing lists).

An online survey was administered through the Google Forms platform. To ensure participation in the study, potential biases related to internet access were taken into account. University of Zaragoza’s own channels, such as the bulletin board, newsletter, and distribution lists, were used to reach participants. A final sample of 458 participants was obtained, all of whom were university employees meeting the inclusion criteria.

### Variables and instruments

The ***dependent variable*** was general health, understood as a comprehensive state of physical, mental, and social well-being, not merely the absence of disease (World Health Organization, 2006). General health was assessed using the General Health Questionnaire (GHQ-12) by Goldberg, in its Spanish validated version [25]. This questionnaire consists of 12 items (six positive and six negative), with responses on a 4-point Likert scale (0–3). The questionnaire shows adequate psychometric properties in previous studies (α = 0.84) [26]. Higher scores indicate greater emotional symptomatology. The internal consistency in our sample was excellent (α = 0.92).

***Independent variables included:***

- **Sociodemographic variables:** Sex, age, marital status, place of residence, cohabitation, number of dependents, type of contract, position, seniority, and intention to quit.
- **Technostress:** Assessed using the Technostress Questionnaire (techno-anxiety and techno-fatigue), designed to identify the negative psychological state associated with the — or anticipated use — of ICT, characterized by a perceived mismatch between technological demands and resources [27]. It consists of 26 items distributed across three dimensions: affective (anxiety and fatigue), attitudinal (scepticism toward technology), and cognitive (inefficacy beliefs). Responses were recorded on a 7-point Likert scale (0 = never to 6 = always). Scores are obtained by averaging the item responses within each dimension, and a total technostress score is computed as the mean of all items, with higher scores indicating greater technostress. The possible range of total scores is 0–6. In accordance with the authors’ recommendations, the construct was treated as a continuous variable in all analyses rather than categorized into thresholds. The questionnaire has demonstrated adequate internal consistency (α > 0.70) in previous studies [27]. The internal consistency in our sample was excellent (α = 0.94).
- **Stress:** Measured by the Perceived Stress Scale (PSS-10), in its Spanish validated version, designed to measure the degree to which life situations are perceived as stressful [28]. It consists of 10 items with a 5-point Likert scale (0 = never to 4 = very often). Higher scores indicate higher perceived stress, with adequate internal consistency in previous studies (α = 0.68-0.82) [28,29]. The internal consistency in our sample was excellent (α = 0.90).
- **Burnout syndrome:** Defined as a response to chronic workplace stress, characterized by emotional exhaustion, cynicism, and low professional efficacy [30]. It was assessed using the Maslach Burnout Inventory-General Survey (MBI-GS) in its Spanish validated version [31]. This generic instrument is useful for measuring burnout across all types of jobs regardless of specific tasks. High scores in exhaustion and cynicism, and low scores in professional efficacy indicate burnout. It consists of 15 items answered on a 7-point Likert scale (0 = never to 6 = always). The questionnaire has adequate psychometric properties in previous studies (α = 0.67-0.85) [31,32]. The internal consistency in our sample was acceptable (α = 0.75).
- **Social support:** Understood as the perceived availability of emotional and instrumental resources from significant relationships. It was measured using the House and Wells questionnaire (1978), which distinguishes between emotional and instrumental support, in its Spanish validated version by National Institute for Safety and Health at Work (INSST) of the Government of Spain [33] and has adequate psychometric properties in previous studies [34,35]. It assesses emotional support (work-related and non-work-related) and instrumental support (work-related). The score for each block is obtained by adding up the values on the scale. Higher scores reflect greater perceived social support, with maximum scores of 15, 12, and 9 points respectively for each type of support. The internal consistency in our sample was good (α = 0.87).
- **Job satisfaction:** Evaluated through the Overall Job Satisfaction Scale by Warr, Cook, and Wall (1979), in its Spanish validated version by INSST of the Government of Spain [36] and has adequate psychometric properties in previous studies [34] (α = 0.85-0.88). The instrument includes 15 items evaluating overall, intrinsic, and extrinsic satisfaction using a 7-point Likert scale (from very dissatisfied to very satisfied). The overall job satisfaction score is obtained additively, by summing the scores of each of the 15 items. The overall job satisfaction questionnaire also allows for the generation of two subscales. Thus, the subscales of intrinsic job satisfaction (by summing the even-numbered items) and extrinsic job satisfaction (by summing the odd-numbered items) were created. Higher scores indicate greater job satisfaction, i.e., a more positive affective response from the worker toward their job and its conditions. The internal consistency in our sample was good (α = 0.89).
- **Work-life areas:** Analysed using the “Six Areas of Worklife Questionnaire – Short Version” validated in Spanish [37]. This instrument includes 18 items distributed across six subscales: manageable workload, control, sense of community, fairness, value congruence, and rewards. Responses are recorded on a 5-point Likert scale (1 = strongly disagree to 5 = strongly agree), where higher scores indicate a more positive evaluation of the respective area. The questionnaire shows adequate psychometric properties in previous studies (α = 0.66 to 0.80, ω = 0.65 to 0.84) [37,38]. The internal consistency in our sample was good (α = 0.85).
- **Resilience:** Measured with the 10-item Connor-Davidson Resilience Scale (CD-RISC 10), in its Spanish validated version with adequate internal consistency (α = 0.87) [39]. The questionnaire uses a 5-point Likert scale from 0 (never) to 4 (almost always). The questionnaire shows adequate psychometric properties in previous studies (α = 0.82) [40]. Higher scores indicate greater adaptability in changing situations and effective coping with stressful events [41]. The internal consistency in our sample was excellent (α = 0.90).
- **Use of new technologies:** Measured using the “Use of Information and Communication Technologies” questionnaire, which has excellent psychometric properties (α = 0.90). The 35-item questionnaire measures three criteria related to knowledge, use, and attitude toward ICT [42]. Notably, since the questionnaire is primarily aimed at teaching staff, they completed it in full. However, administrative and service staff only completed the first eight questions related to the Knowledge criterion. The adapted questionnaire shows adequate psychometric properties in previous studies (α = 0.83) [43]. Responses are recorded on a Likert scale, with higher scores indicating higher levels of knowledge, use, and positive attitude toward ICT. The internal consistency in our sample was good (α = 0.89).

### Ethical considerations

The study was approved by the Aragón Ethics Committee (CEICA, PI22-259) and by the Data Protection Unit of the University of Zaragoza (RAT 2022-93), complying with the Declaration of Helsinki and the national Organic Law 3/2018 on data protection. Participation was voluntary, anonymous, and preceded by electronic informed consent. The right to withdraw at any time was guaranteed. The research did not require funding or institutional resources with costs.

### Data analysis

Firstly, to describe the sample composition, a descriptive analysis was performed using frequencies for categorical variables and means with standard deviations for continuous variables. The normality of the data was primarily assessed using the Shapiro-Wilk test, chosen for its greater power in detecting deviations from normality, particularly in small to moderate sample sizes [44]. Results indicated that the data did not follow a normal distribution. Complementary visual assessments were conducted using Q-Q plots and histograms to further examine the distribution of variables.

Secondly, Spearman’s rank-order correlation coefficients were computed to evaluate the relationships between GHQ-12 scores and various independent variables, including ordinal, non-normally distributed continuous, and dichotomous variables (e.g., technostress, burnout), with dichotomous variables coded as binary indicators. To investigate whether these associations varied by employment characteristics, correlations were also calculated separately for subgroups defined by academic role, distinguishing between teaching/research and administrative personnel (ARS vs. ASS). Contract type (permanent vs. temporary/other) and organizational tenure (<12 years vs. ≥ 12 years) differences were measured as well. Given the exploratory and hypothesis-generating nature of these bivariate and subgroup correlations, no formal correction for multiple comparisons was applied. Accordingly, p-values from these analyses should be interpreted with caution, and greater emphasis is placed on the size and direction of the associations (Spearman’s ρ) and on the consistency of patterns across subgroups and models.

Thirdly, to address the main objective of identifying general health predictors, a multiple linear regression analysis was conducted [45]. For the main and subgroup regression models, candidate predictors were selected following a theory-driven and data-driven screening strategy. Specifically, for each subgroup we considered all sociodemographic, psychosocial and occupational variables that showed a significant bivariate association with GHQ-12 (p ≤ .05) in the corresponding correlation matrix, together with their theoretical relevance as psychosocial risk factors. The selected predictors were then entered simultaneously using the ENTER method. For each predictor, both unstandardized (B) and standardized (β) coefficients are reported, together with 95% confidence intervals for B. In the regression models, interpretation prioritises effect sizes (standardised coefficients β) and 95% confidence intervals over p-values alone. Given that the normality assumption of residuals was not fully met based on the Shapiro-Wilk test, a bootstrapping procedure with 1,000 resamples and percentile-based 95% confidence intervals was applied to enhance the robustness of the regression estimates. This approach allowed for more reliable inference regarding the significance of predictors and the precision of confidence intervals. The significance level was set at p < 0.05. All analyses were carried out using SPSS version 25.0 [46]. There were no missing data in the dataset.

## Results

A total of 458 workers from the University of Zaragoza participated in the study. Regarding sociodemographic characteristics, 52.4% of the participants were women, with an average age of 49 years. The vast majority (90.4%) resided in Zaragoza, and 74% were married or living with a partner. In terms of employment characteristics, 59.2% held a permanent contract or were civil servants, 19.9% held a temporary contract, and 18.8% held an interim contract. Additionally, 60.3% of participants had been employed at the University of Zaragoza for more than 12 years, and 50.2% did not intend to leave their job within the next five years. Among the respondents, 56.3% were Academic and Research Staff (ARS), while 43.7% were Administrative and Service Staff (ASS) (see Table 1).

**Table 1:** Correlation between sociodemographic variables and health.

Variables	Participants (n = 458) n (%)	GHQ-12 Mean (SD)	GHQ-12 Median (IQR)	GHQ-12 (R)	p-value	95% Confidence Intervals
**Age**						
Y and Z generation, n (%)	106 (23.1)	12.59 (6.65)	11.00 (8)	.058	.212	−0.036 to 0.152
X generation, n (%)	352 (76.9)	13.10 (6.26)	12.00 (7)			
**Gender**						
Female, n (%)	240 (52.4)	13.07 (6.12)	12.00 (8)	−0.041	.384	−0.135 to 0.054
Male, n (%)	218 (47.6)	12.89 (6.61)	11.00 (7)			
**Marital status**						
With a partner, n (%)	339 (74)	12.74 (6.27)	11.00 (8)	.079	.090	−0.015 to 0.172
Without a partner, n (%)	119 (26)	13.66 (6.56)	12.00 (7)			
**In-home coexistence**						
Lives alone, n (%)	62 (13.5)	13.66 (6.08)	12.00 (7)	−0.070	.133	−0.164 to 0.024
Lives with others, n (%)	396 (86.5)	12.88 (6.39)	11.00 (8)			
**Seniority at work**						
Less than 12 years, n (%)	182 (39.7)	12.20 (6.13)	11.00 (7)	0.116*	.013	0.022 to 0.208
12 years or more, n (%)	276 (60.3)	13.50 (6.46)	12.00 (8)			
**Type of employment contract**						
Permanent contract, n (%)	271 (59.2)	13.46 (6.32)	12.00 (8)	−0.121*	.010	−0.213 to −0.027
Temporary contract or other, n (%)	187 (40.8)	12.29 (6.36)	11.00 (7)			
**Employment category**						
Academic and Research Staff (ARS), n (%)	258 (56.3)	13.68 (6.59)	12.00 (8)	−0.115*	.014	−0.207 to −0.021
Administrative and Service Staff (ASS), n (%)	200 (43.7)	12.09 (5.93)	11.00 (7)			
**Intention to leave within 5 years**						
Yes, n (%)	73 (15.9)	13.45 (6.82)	12.00 (8)	−0.023	.623	−0.117 to 0.071
No, n (%)	385 (84.1)	12.89 (6.26)	11.00 (8)			

*Note.* Significant differences (p ≤ .05) are highlighted in bold. *R*, Spearman’s Rho; GHQ-12, General Health Questionnaire. *: The correlation is significant at the 0.05 level (two-tailed).

Regarding differences in general health based on sociodemographic and employment variables, Table 1 shows that significant differences were found only in relation to seniority, contract type (temporary vs. permanent), and employment category (ARS vs. ASS). Consequently, regression models were developed using these six variables. Moreover, having more than 12 years of tenure was associated with **poorer** health (i.e., higher GHQ-12 scores), whereas holding a temporary contract and being part of the ARS group were also linked to poorer health and a higher prevalence of emotional symptoms.

Regarding the psychological variables (Table 2), all showed significant correlations with health in the expected direction, except for knowledge and use of ICT, which did not exhibit a significant correlation.

**Table 2:** Correlation between psychosocial variables and health.

Variables	Participants (n = 458) n (%)	GHQ-12 Mean (SD)	GHQ-12 Median (IQR)	GHQ-12 (*R*)	p-value	95% Confidence Intervals	
						Lower	Upper
**Total sample**	458 (100)	12.98 (6.35)	12 (7)	**–**	**–**	**–**	**–**
**Technostress**				0.450**	<.001	.371	.522
Yes*, n (%)*	140 (30.6)	17.16 (6.70)	16 (10)				
No*, n (%)*	318 (69.4)	11.14 (5.23)	10 (5)				
**Technofatigue**				0.451**	<.001	.373	.523
Yes*, n (%)*	145 (31.7)	17.03 (6.71)	16 (10)				
No*, n (%)*	313 (68.3)	11.11 (5.21)	10 (5)				
**Technoanxiety**				0.445**	<.001	.366	.517
Yes*, n (%)*	152 (33.2)	16.82 (6.76)	15 (9)				
No*, n (%)*	306 (66.8)	11.08 (5.18)	10 (5)				
**Perceived Stress** *, M (SD)*	17.66 (8.11)	–	–	0.767**	<.001	.725	.803
**Burnout**				.452**	<.001	.374	.524
Yes*, n (%)*	115 (25.1)	17.91 (6.68)	17 (11)				
No*, n (%)*	343 (74.9)	11.33 (5.31)	10 (5)				
**Exhaustion**				.610**	<.001	.548	.666
Yes*, n (%)*	225 (49.1)	16.64 (6.43)	16 (9)				
No*, n (%)*	233 (50.9)	9.45 (3.76)	9 (4)				
**Cynicism**				.485**	<.001	.409	.554
Yes*, n (%)*	271 (59.2)	15.38 (6.69)	14 (10)				
No*, n (%)*	187 (40.8)	9.51 (3.70)	9 (4)				
**Efficiency**				−.432**	<.001	−.506	−.352
Yes*, n (%)*	284 (62)	16.18 (6.59)	15 (10)				
No*, n (%)*	174 (38)	11.02 (5.33)	10 (6)				
**Social-emotional support**							
Work-related*, M (SD)*	10.52 (4.09)	–	–	−0.341**	<.001	−.422	−.255
Non-work-related*, M (SD)*	8.44 (3.34)	–	–	−0.194**	<.001	−.283	−.101
**Instrumental Social Support** *, M (SD)*	6.11 (2.52)	–	–	−0.296**	<.001	−.379	−.207
**Overall job satisfaction** *, M (SD)*	70.41 (17.21)	–	–	−0.399**	<.001	−.475	−.316
Extrinsic*, M (SD)*	37.29 (9.19)	–	–	−0.345**	<.001	−.425	−.259
Intrinsic*, M (SD)*	33.11 (8.85)	–	–	−0.416**	<.001	−.491	−.335
**Resilience** *, M (SD)*	28.3 (6.80)	–	–	−0.508**	<.001	−.575	−.435
**Areas of working life**							
Manageable work overload*, M (SD)*	8.60 (3.21)	–	–	−0.365**	<.001	−.444	−.280
Control*, M (SD)*	9.81 (2.32)	–	–	−0.349**	<.001	−.429	−.264
Rewards*, M (SD)*	10.21 (2.81)	–	–	−0.372**	<.001	−.451	−.288
Justice*, M (SD)*	8.31 (2.49)	–	–	−0.262**	<.001	−.348	−.172
Value congruence*, M (SD)*	8.97 (2.83)	–	–	−0.315**	<.001	−.398	−.228
Sense of Community*, M (SD)*	11.10 (3.06)	–	–	−0.262**	<.001	−.348	−.172
**ICT**							
Knowledge*, M (SD)*	14.91 (3.87)	–	–	−0.50	.281	−.144	.044
Usage*, M (SD)*	17.10 (6.94)	–	–	−0.018	.780	−.144	.109
Attitudinal*, M (SD)*	28.26 (8.59)	–	–	−0.263**	<.001	−.377	−.141

*Note.* Significant differences (p ≤ .05) are highlighted in bold. *R*, Spearman’s Rho; M: mean; SD: standard deviation; IQR: Interquartile Range; GHQ-12: General Health Questionnaire. *: The correlation is significant at the 0.05 level (two-tailed). **: The correlation is significant at the 0.01 level (two-tailed).

Across all six models, GHQ-12 scores were significantly and positively correlated with technostress, perceived stress, and burnout (see Table 3). Technostress showed moderate associations, with the strongest correlation observed in the ≥ 12 years group (ρ = .528, p < .001) and the weakest in the Temporary/Others group (ρ = .357, p < .001). Perceived stress (PSS) exhibited the strongest and most consistent correlations across all models, ranging from ρ = .741 (p < .001) in the < 12 years group to ρ = .789 (p < .001) in the ≥ 12 years group. Burnout (MBI-GS total) was also significantly associated with emotional symptoms, with correlation coefficients ranging from ρ = .392 (p < .001) in the Temporary/Others group to ρ = .491 (p < .001) in the ≥ 12 years group.

**Table 3:** Correlation between health (GHQ-12) and sociodemographic and psychosocial variables and job variables.

Variable	ARS GHQ-12 (*R*)	p-value	ASS GHQ-12 (*R*)	p-value	Permanent GHQ-12 (*R*)	p-value	Temp./Others GHQ-12 (*R*)	p-value	<12 years GHQ-12 (*R*)	p-value	≥12 years GHQ-12 (*R*)	p-value
Sex	–.051	.413	–.057	.422	–.048	.427	–.049	.504	–.040	.593	–.042	.486
Age	.020	.753	.184**	.009	.009	.878	.031	.676	–.029	.701	.035	.564
Marital status	.064	.307	.114	.109	.106	.082	.080	.279	.118	.112	.061	.315
Living arrangement	–.034	.582	–.126	.076	–.050	.409	–.104	.157	–.064	.390	–.069	.251
Intention to quit	.023	.717	–.079	.265	.059	.333	–.129	.078	–.150*	.044	.068	.262
Technostress (fatigue & anxiety)	.486**	<.001	.403**	<.001	.515**	<.001	.357**	<.001	.335**	<.001	.528**	<.001
Perceived stress (PSS)	.768**	<.001	.765**	<.001	.783**	<.001	.757**	<.001	.741**	<.001	.789**	<.001
Burnout (MBI-GS total)	.464**	<.001	.429**	<.001	.490**	<.001	.392**	<.001	.390**	<.001	.491**	<.001
Emotional support – work-related	–.337**	<.001	–.337**	<.001	–.305**	<.001	–.392**	<.001	–.379**	<.001	–.313**	<.001
Emotional support – non-work-related	–.150*	.016	–.253**	<.001	–.234**	<.001	–.135	.066	–.158*	.034	–.213**	<.001
Instrumental support	–.301**	<.001	–.244**	<.001	–.264**	<.001	–.320**	<.001	–.282**	<.001	–.286**	<.001
Job satisfaction (SL total)	–.395**	<.001	–.395**	<.001	–.387**	<.001	–.448**	<.001	–.462**	<.001	–.369**	<.001
Resilience (CD-RISC total)	–.532**	<.001	–.485**	<.001	–.528**	<.001	–.449**	<.001	–.420**	<.001	–.545**	<.001
Manageable workload	–.350**	<.001	–.363**	<.001	–.332**	<.001	–.385**	<.001	–.377**	<.001	–.339**	<.001
Control over work	–.372**	<.001	–.340**	<.001	–.372**	<.001	–.322**	<.001	–.313**	<.001	–.364**	<.001
Sense of community	–.244**	<.001	–.268**	<.001	–.288**	<.001	–.229**	.002	–.250**	<.001	–.272**	<.001
Organizational justice	–.294**	<.001	–.228**	.001	–.252**	<.001	–.287**	<.001	–.277**	<.001	–.248**	<.001
Value congruence	–.314**	<.001	–.299**	<.001	–.341**	<.001	–.258**	<.001	–.314**	<.001	–.298**	<.001
Rewards	–.413**	<.001	–.323**	<.001	–.406**	<.001	–.350**	<.001	–.364**	<.001	–.390**	<.001
ICT – Knowledge	–.078	.211	–.076	.285	–.071	.246	–.010	.888	.100	.179	–.133*	.027
ICT – Use	–.028	.655	—	—	–.062	.427	.050	.638	.014	.888	–.055	.504
ICT – Attitudinal (ARS only)	–.266**	<.001	—	—	–.246**	.001	–.258*	.014	–.271**	.005	–.257**	.002

*Note. R*, Spearman’s Rho; *: The correlation is significant at the 0.05 level (two-tailed). **: The correlation is significant at the 0.01 level (two-tailed). GHQ-12, General Health Questionnaire. ARS = Teaching & Research Staff; ASS = Administrative Staff; Permanent = permanent contracts; Temp./Others = temporary or other contract types; <12 years = participants with less than 12 years of service; ≥ 12 years = participants with 12 years or more.

Work-related emotional support showed significant negative correlations across all subgroups, strongest among Temporary/Others (ρ = –.392, p < .001) and weakest in the Permanent group (ρ = –.305, p < .001). Non-work-related emotional support was also inversely associated, though the correlation was weaker and non-significant in the Temporary/Others group; the strongest association was observed among ASS staff (ρ = –.253, p < .001). Instrumental social support negatively correlated with GHQ-12 scores in all models, with coefficients ranging from ρ = –.244 (p < .001) in ASS to ρ = –.320 (p < .001) in the Temporary/Others group.

Job satisfaction was inversely related to emotional distress in every model, with the highest correlation in the < 12 years group (ρ = –.462, p < .001) and the lowest among Permanent employees (ρ = –.387, p < .001). Resilience showed strong negative associations across all groups, ranging from ρ = –.449 (p < .001) in Temporary/Others to ρ = –.545 (p < .001) among those with ≥12 years of service.

All six Areas of Worklife variables were inversely related to emotional distress: manageable workload (ρ = –.332 to –.385, p < .001), perceived control (ρ = –.313 to –.372, p < .001), sense of community (ρ = –.229 to –.288, p ≤ .002), organizational justice (ρ = –.228 to –.294, p ≤ .001), value congruence (ρ = –.258 to –.341, p < .001), and rewards (ρ = –.323 to –.413, p < .001). The intention to quit showed a weak but significant, negative correlation only in the < 12 years group (ρ = –.150, p = .044).

Finally, attitudinal ICT competence was negatively correlated with GHQ-12 scores among ARS staff (ρ = –.266, p < .001), and ICT knowledge showed a small but significant negative correlation only in the ≥ 12 years group (ρ = –.133, p = .027). No significant correlations were found for sex, age (except in ASS: ρ = .184, p = .009), marital status, living arrangements, or ICT usage in any of the models.

Next, the results of the analysis on health models are presented. In this study, health is defined as a state of overall well-being characterized by the absence of emotional symptomatology.

A multiple linear regression analysis was conducted to identify predictors of emotional symptomatology (GHQ-12) among the university’s Academic and Research Staff (ARS) (see Table 4). The overall model was statistically significant, F(15, 237) = 28.80, p < .001, explaining approximately 64.6% of the variance in GHQ-12 scores (adjusted R^2^ = .623). The strongest predictors of increased emotional symptomatology were higher levels of perceived stress (β = .503, p < .001) and technostress (β = .166, p < .001). Conversely, greater resilience (β = –.196, p < .001) and higher levels of non-work-related emotional support (β = –.096, p = .027) were significant protective factors. Other variables, such as emotional work-related support, burnout, job satisfaction, and value congruence, did not significantly predict emotional symptomatology in the final model.

**Table 4:** Health model in Academic and Research Staff (ARS).

Predictor	B	SE	β	t	p	95% CI (B)	Tolerance	VIF
(Constant)	14.003	2.724	—	5.141	<.001	[8.637, 19.368]	—	—
Technostress	2.328	0.612	.166	3.805	<.001	[1.123, 3.534]	.783	1.277
Perceived Stress	0.399	0.044	.503	9.042	<.001	[0.312, 0.486]	.482	2.074
Burnout	0.220	0.723	.015	0.305	.761	[–1.203, 1.644]	.611	1.637
Emotional Support	–0.153	0.141	–.094	–1.087	.278	[–0.431, 0.125]	.200	4.997
Emotional Support (Non-work-related)	0.186	0.084	.096	2.223	.027	[0.021, 0.351]	.807	1.240
Instrumental Support	–0.128	0.216	–.049	–0.592	.554	[–0.554, 0.298]	.221	4.519
Job Satisfaction	0.000	0.025	.000	0.007	.994	[–0.050, 0.050]	.344	2.907
Resilience	–0.183	0.051	–.196	–3.591	<.001	[–0.284, –0.083]	.503	1.987
Manageable Workload	–0.134	0.109	–.058	–1.236	.218	[–0.348, 0.080]	.682	1.466
Control	–0.015	0.147	–.005	–0.101	.919	[–0.304, 0.274]	.578	1.729
Sense of Community	0.160	0.110	.075	1.453	.148	[–0.057, 0.378]	.555	1.802
Justice	–0.065	0.131	–.025	–0.495	.621	[–0.323, 0.194]	.583	1.715
Value Congruence	–0.015	0.117	–.007	–0.132	.895	[–0.246, 0.215]	.560	1.787
Rewards	–0.087	0.124	–.037	–0.704	.482	[–0.332, 0.157]	.541	1.848
ICT Attitude	–0.059	0.034	–.078	–1.744	.082	[–0.126, 0.008]	.750	1.332

*Note*. Dependent variable: health (GHQ-12). Higher scores indicate poorer health and a greater degree of emotional symptomatology.

Next, a multiple linear regression analysis was conducted to identify predictors of emotional symptomatology (GHQ-12) among Administrative and Service Staff (ASS) (Table 5). The overall model was statistically significant, F(15, 184) = 20.78, p < .001, explaining approximately 63% of the variance in emotional symptoms (adjusted R^2^ = .599). Significant predictors included perceived stress (β = .579, p < .001) and technostress (β = .111, p = .035), both positively associated with increased emotional symptomatology, while resilience (β = –.166, p = .004) was significantly associated with lower levels of emotional symptoms.

**Table 5:** Health model in Administrative and Service Staff (ASS).

Predictor	B	SE	β	t	p	95% CI (B)	Tolerance	VIF
(Constant)	8.336	3.480	–	2.395	.018	[1.470, 15.203]	–	–
Age	0.573	0.814	0.035	0.703	.483	[-1.034, 2.179]	0.836	1.196
Technostress	1.443	0.678	0.111	2.128	.035	[0.105, 2.781]	0.746	1.340
Perceived stress	0.431	0.045	0.579	9.628	<.001	[0.343, 0.519]	0.558	1.791
Burnout	1.100	0.839	0.077	1.310	.192	[-0.557, 2.756]	0.585	1.711
Emotional support (work)	−0.127	0.159	−0.084	−0.797	.427	[-0.441, 0.187]	0.183	5.460
Emotional support (non-work)	0.085	0.087	0.048	0.981	.328	[-0.086, 0.257]	0.842	1.187
Instrumental support	0.257	0.255	0.103	1.010	.314	[-0.245, 0.760]	0.195	5.125
Job Satisfaction	−0.040	0.030	−0.117	−1.354	.177	[-0.098, 0.018]	0.269	3.719
Resilience	−0.150	0.051	−0.166	−2.952	.004	[-0.250, -0.050]	0.637	1.570
Manageable workload	−0.099	0.101	−0.052	−0.976	.330	[-0.298, 0.101]	0.724	1.381
Control	−0.013	0.152	−0.005	−0.083	.934	[-0.313, 0.288]	0.540	1.852
Sense of community	−0.014	0.128	−0.007	−0.108	.914	[-0.266, 0.238]	0.480	2.081
Justice	−0.173	0.152	−0.071	−1.144	.254	[-0.473, 0.126]	0.520	1.922
Value congruence	0.112	0.138	0.051	0.813	.417	[-0.160, 0.385]	0.506	1.977
Rewards	0.239	0.146	0.115	1.637	.103	[-0.049, 0.526]	0.410	2.437

*Note*. Dependent variable: health (GHQ-12). Higher scores indicate poorer health and a greater degree of emotional symptomatology.

A multiple linear regression was conducted to examine the predictors of emotional symptomatology (GHQ-12) separately for participants with permanent contracts and those with temporary or other types of contracts. For individuals with permanent contracts (Table 6), the overall model was significant, F(15, 148) = 27.11, p < .001, explaining approximately 73.3% of the variance in emotional symptomatology (adjusted R^2^ = .706).

**Table 6:** Health model in participants with permanent contracts.

Predictor	B	SE	β	t	p	95% CI (B)	Tolerance	VIF
(Constant)	13.190	3.013	—	4.378	<.001	[7.236, 19.144]	—	—
Technostress	3.131	0.696	0.226	4.498	<.001	[1.755, 4.506]	0.717	1.396
Perceived stress	0.386	0.049	0.499	7.837	<.001	[0.289, 0.483]	0.445	2.245
Burnout	0.505	0.829	0.035	0.610	0.543	[-1.133, 2.144]	0.536	1.867
Social-emotional support (work-related)	−0.154	0.165	−0.097	−0.931	0.353	[-0.481, 0.173]	0.167	6.004
Social-emotional support (non-work-related)	0.213	0.088	0.113	2.408	0.017	[0.038, 0.387]	0.818	1.223
Instrumental Social Support	−0.068	0.266	−0.026	−0.256	0.798	[-0.594, 0.458]	0.173	5.767
Overall job satisfaction	0.068	0.032	0.179	2.121	0.036	[0.005, 0.131]	0.252	3.967
Resilience	−0.173	0.059	−0.188	−2.961	0.004	[-0.289, -0.058]	0.449	2.228
Manageable workload	−0.345	0.126	−0.146	−2.743	0.007	[-0.594, -0.097]	0.638	1.568
Control	0.021	0.172	0.008	0.122	0.903	[-0.319, 0.361]	0.475	2.104
Sense of community	0.064	0.126	0.030	0.508	0.612	[-0.185, 0.313]	0.502	1.992
Justice	−0.150	0.150	−0.057	−1.000	0.319	[-0.446, 0.146]	0.550	1.820
Value congruence	−0.060	0.133	−0.027	−0.452	0.652	[-0.322, 0.202]	0.505	1.982
Rewards	−0.207	0.136	−0.090	−1.529	0.129	[-0.475, 0.061]	0.518	1.930
ICT Attitude	−0.073	0.040	−0.093	−1.822	0.071	[-0.153, 0.006]	0.697	1.434

*Note*. Dependent variable: health (GHQ-12). Higher scores indicate poorer health and a greater degree of emotional symptomatology.

Perceived stress was a significant positive predictor of emotional symptoms (β = 0.499, p < .001), indicating that higher stress levels were associated with greater emotional symptomatology. Likewise, technostress was positively and significantly related to symptoms (β = 0.226, p < .001). Resilience showed a significant negative association with emotional symptomatology (β = −0.188, p = .004), suggesting that higher resilience predicted fewer symptoms. Manageable workload also predicted lower emotional symptom levels (β = −0.146, p = .007).

Interestingly, emotional support unrelated to work was positively associated with emotional symptoms (β = 0.113, p = .017), as was general job satisfaction (β = 0.179, p = .036). Other variables—including emotional work-related support, instrumental support, control, justice, value congruence, rewards, and technological aptitude—were not statistically significant predictors in this model.

Thereafter, a multiple linear regression analysis was conducted to identify the predictors of emotional symptomatology (GHQ-12) among participants with temporary or other non-permanent contracts (Table 7). The overall model was statistically significant, F(14, 75) = 8.17, p < .001, explaining approximately 60.4% of the variance in GHQ scores (adjusted R^2^ = .530).

**Table 7:** Health model in participants with temporary or other non-permanent contracts.

Predictor	B	SE	β	t	p	95% CI (B)	Tolerance	VIF
(Constant)	14.696	5.397	—	2.723	.008	[3.945, 25.447]	—	—
Technostress	0.835	1.178	.058	0.708	.481	[-1.513, 3.183]	0.781	1.280
Perceived Stress	0.459	0.085	.551	5.395	<.001	[0.289, 0.628]	0.506	1.976
Burnout	−0.095	1.423	−.006	−0.066	.947	[-2.929, 2.740]	0.622	1.608
Emotional Work Support	−0.458	0.269	−.252	−1.704	.093	[-0.993, 0.078]	0.241	4.142
Instrumental Support	−0.033	0.396	−.011	−0.084	.933	[-0.823, 0.756]	0.292	3.423
General Satisfaction	−0.119	0.052	−.291	−2.292	.025	[-0.222, -0.016]	0.328	3.053
Resilience	−0.102	0.097	−.101	−1.057	.294	[-0.295, 0.091]	0.582	1.720
Manageable Workload	0.194	0.206	.087	0.942	.349	[-0.216, 0.603]	0.625	1.600
Control Possibilities	−0.281	0.270	−.091	−1.040	.301	[-0.820, 0.257]	0.694	1.441
Sense of Community	0.213	0.209	.097	1.018	.312	[-0.204, 0.629]	0.584	1.713
Justice	−0.158	0.256	−.062	−0.616	.540	[-0.667, 0.352]	0.518	1.932
Values Congruence	0.260	0.253	.108	1.027	.308	[-0.244, 0.764]	0.476	2.100
Rewards	0.404	0.270	.163	1.499	.138	[-0.133, 0.941]	0.447	2.237
ICT Attitude	−0.006	0.062	−.008	−0.099	.922	[-0.130, 0.118]	0.796	1.257

*Note*. Dependent variable: health (GHQ-12). Higher scores indicate poorer health and a greater degree of emotional symptomatology.

Perceived stress emerged as the strongest and only statistically significant predictor (β = .551, p < .001), indicating that higher perceived stress levels were associated with greater emotional symptomatology. Job satisfaction was also a significant negative predictor (β = –.291, p = .025), suggesting that higher job satisfaction was linked to fewer symptoms.

Although emotional work-related support approached significance (β = –.252, t(75) = –1.70, p = .093), it did not meet the conventional threshold for significance. All other predictors—including technostress, resilience, control, and organizational justice—did not significantly contribute to the model.

Furthermore, for employees with less than 12 years of seniority (Table 8), the model was statistically significant, F(16, 89) = 7.22, p < .001, explaining approximately 56.5% of the variance in GHQ-12 scores (adjusted R^2^ = .487). Among all predictors, perceived stress was the only statistically significant variable (β = 0.533, p < .001), indicating that higher perceived stress levels were associated with poorer mental health.

**Table 8:** Health model in participants with tenure of less than 12 years.

Predictor	B	SE	β	t	p	95% CI (B)	Tolerance	VIF
(Constant)	13.993	5.969	–	2.344	.021	[2.132, 25.854]	–	–
Intention to leave	−0.320	1.634	−0.015	−0.196	.845	[-3.567, 2.926]	.826	1.210
Technostress	1.145	1.029	0.086	1.113	.269	[-0.900, 3.190]	.817	1.224
Perceived stress	0.456	0.083	0.533	5.514	<.001	[0.292, 0.621]	.524	1.910
Burnout	−0.619	1.272	−0.043	−0.487	.628	[-3.146, 1.908]	.614	1.629
Emotional support (work-related)	−0.445	0.265	−0.260	−1.679	.097	[-0.972, 0.082]	.204	4.908
Emotional support (non-work-related)	0.140	0.160	0.072	0.877	.383	[-0.178, 0.458]	.730	1.369
Instrumental support	0.019	0.386	0.007	0.050	.960	[-0.748, 0.787]	.266	3.758
Job satisfaction	−0.068	0.045	−0.183	−1.498	.138	[-0.157, 0.022]	.329	3.042
Resilience	−0.137	0.095	−0.129	−1.443	.152	[-0.325, 0.051]	.614	1.630
Manageable workload	0.114	0.186	0.052	0.614	.541	[-0.255, 0.483]	.684	1.461
Control possibilities	−0.168	0.250	−0.057	−0.670	.505	[-0.665, 0.330]	.673	1.487
Sense of community	0.245	0.191	0.117	1.286	.202	[-0.134, 0.624]	.590	1.696
Perceived justice	−0.145	0.240	−0.059	−0.605	.546	[-0.622, 0.332]	.508	1.968
Value congruence	0.020	0.226	0.009	0.089	.929	[-0.429, 0.469]	.513	1.950
Rewards	0.402	0.241	0.172	1.671	.098	[-0.076, 0.881]	.460	2.174
ICT Attitude	−0.055	0.056	−0.080	−0.972	.334	[-0.167, 0.057]	.730	1.370

*Note*. Dependent variable: health (GHQ-12). Higher scores indicate poorer health and a greater degree of emotional symptomatology.

No other predictors reached statistical significance, although work-related emotional support (β = –0.260, p = .097) and rewards (β = 0.172, p = .098) showed marginal trends toward significance.

Then, for employees with 12 years or more of seniority (Table 9), the model was statistically significant, F(16, 131) = 25.19, p < .001, explaining 75.5% of the variance in GHQ-12 scores (R^2^ = .755, adjusted R^2^ = .725). Perceived stress was the strongest positive predictor of emotional symptoms (β = .473, p < .001), followed by technostress, which also showed a significant positive association (β = .235, p < .001). Conversely, resilience was significantly and negatively associated with emotional symptoms (β = –.212, p = .002), indicating that higher resilience corresponds to fewer emotional difficulties.

**Table 9:** Health model in participants with tenure of 12 years or more.

Predictor	B	SE	β	t	p	95% CI (B)	Tolerance	VIF
(Constant)	13.135	3.490	–	3.764	<.001	[6.231, 20.039]	–	–
Technostress	3.406	0.789	.235	4.318	<.001	[1.846, 4.966]	.635	1.576
Perceived stress	0.362	0.051	.473	7.069	<.001	[0.260, 0.463]	.418	2.394
Burnout	0.371	0.894	.025	0.415	.679	[-1.397, 2.139]	.520	1.924
Social-emotional support (work-related)	−0.073	0.167	−.045	−0.437	.663	[-0.404, 0.258]	.174	5.740
Social-emotional support (non work-related)	0.214	0.095	.110	2.241	.027	[0.025, 0.403]	.775	1.290
Instrumental Social Support	−0.167	0.268	−.065	−0.623	.534	[-0.698, 0.363]	.174	5.744
Overall job satisfaction	0.045	0.034	.111	1.327	.187	[-0.022, 0.111]	.267	3.749
Resilience	−0.188	0.059	−.212	−3.169	.002	[-0.306, -0.071]	.420	2.380
Manageable workload	−0.327	0.135	−.135	−2.417	.017	[-0.595, -0.059]	.597	1.676
Control possibilities	0.073	0.179	.025	0.408	.684	[-0.280, 0.426]	.480	2.082
Sense of community	0.057	0.134	.027	0.426	.671	[-0.208, 0.323]	.480	2.082
Perceived justice	−0.099	0.157	−.037	−0.631	.529	[-0.409, 0.211]	.552	1.811
Value congruence	0.003	0.142	.002	0.024	.981	[-0.278, 0.285]	.470	2.127
Rewards	−0.290	0.147	−.120	−1.969	.051	[-0.581, 0.001]	.501	1.995
ICT Knowledge	0.081	0.102	.040	0.794	.429	[-0.121, 0.284]	.749	1.335
ICT Attitude	−0.055	0.046	−.066	−1.189	.236	[-0.146, 0.036]	.605	1.654

*Note*. Dependent variable: health (GHQ-12). Higher scores indicate poorer health and a greater degree of emotional symptomatology.

A manageable workload was also negatively related to emotional symptoms (β = –.135, p = .017), while non-work-related emotional support was positively associated with emotional symptoms (β = .110, p = .027). Other variables — including burnout, work-related emotional support, instrumental support, life satisfaction, perceived control, sense of community, organizational justice, rewards, value congruence, digital knowledge, and attitudinal skills — were not significant predictors.

## Discussion

This study aimed to analyse the levels of technostress among university staff and its relationship with health, within a context where technological overexposure and constant connectivity have a significant impact on psychosocial risks and people’s well-being [47]. Additionally, it sought to explore the relationships among technostress, health, and a range of psychological, psychosocial, and occupational variables.

The results show significant differences in health depending on organizational seniority, contract type, and job position. University employees with more than 12 years of seniority, permanent contracts, and those in Academic and Research Staff (ARS) positions reported poorer health levels. These findings align with previous research indicating that faculty members face greater work-related stress and negative consequences, particularly those working in on-site institutions, with higher age and experience [48,49].

Regarding organizational seniority, descriptive analyses showed that employees with 12 years or more of service reported poorer health, as reflected in higher GHQ-12 scores, compared with those with less tenure. The health model for long-tenured employees also demonstrated a greater explanatory capacity for health outcomes (adjusted R^2^ = 0.725 vs. 0.487), suggesting a stronger influence of the organizational and psychosocial context on this group. In particular, lower scores on manageable workload (i.e., greater work overload) emerged as a key factor linked to physical and mental health deterioration among long-tenured staff [50–53]. These findings challenge the intuitive assumption that longer tenure and job stability are necessarily protective and instead point to the potential cumulative impact of chronic psychosocial demands and technostress on health.

Concerning contract type, the health model for permanent employees showed greater explanatory capacity compared to those with temporary or other non-permanent contracts. Notably, the explanatory variables coincided with those observed in the group with greater seniority, which is consistent with the Spanish university system, where seniority is often linked to job stability. Temporary contracts are typical during the early career stages, involving younger workers as well as those on scholarships and research contracts [54–57].

Regarding job position, both ARS and ASS in Spanish universities exhibit sociodemographic profiles similar to those at the University of Zaragoza, where employees mainly range from 50 to 59 years old, mostly with permanent contracts, especially after the age of 50. ASS are predominantly female, whereas ARS are mostly male [57]. Thus, the health models for both groups showed comparable predictive capacity.

In all health models, perceived stress played a significant explanatory role. Previous research has documented moderate stress levels among university staff [58,59]. Traditionally, ARS showed lower stress levels than ASS, but recent organizational and technological changes have introduced new stressors [60,61]. Specifically, men in stable jobs tend to experience lower stress, where teaching experience and seniority foster coping strategies such as cognitive reappraisal and self-efficacy [62].

In the models for employees with greater seniority and permanent contracts — as well as by job position —, technostress and resilience also emerged as relevant variables. Previous studies confirm that university faculty experience higher levels of technostress than technical and administrative staff, particularly in on-site universities and among older, more experienced faculty members [63,64]. This phenomenon is linked to higher ICT demands, lack of clear guidelines, and feelings of inefficacy [64,65]. Conversely, resilience has been confirmed as a protective factor against health deterioration, consistent with studies highlighting its buffering role against stress, technostress, and burnout [66,67]. Individuals with low resilience are more vulnerable to stress’s negative effects and exhibit greater emotional vulnerability, manifesting symptoms such as anxiety, depression, impulsivity, or low self-esteem [68–70].

Moreover, job satisfaction emerged as a key predictor of health, particularly in relation to contract type. This finding aligns with the literature linking higher job satisfaction to better mental health, fewer depressive and anxious symptoms, greater self-esteem, and improved physical health [71–73].

Finally, non-work-related emotional social support was identified as a significant health predictor among employees with longer seniority, temporary contracts, and those in the ARS group. This type of social support is important in predicting health and well-being among university workers and other sectors. Greater emotional support from family and friends is associated with better mental health, lower stress, greater overall well-being, and higher life satisfaction and morale [74–76]. However, it is also important to note that, while non-work-related emotional social support showed the expected negative bivariate association with emotional symptoms, in some adjusted subgroup models it emerged as a small positive predictor. Given that this variable behaves as a protective factor at the correlational level and only becomes positive in highly saturated models including a broad set of correlated psychosocial predictors, this counterintuitive pattern is best interpreted as reflecting suppression and construct overlap rather than a genuinely detrimental effect of extra-work emotional support on health. Accordingly, this finding should be viewed with caution and understood as an indication of the complex way in which external emotional resources are mobilised under conditions of high distress, rather than as evidence that extra-work emotional support worsens workers’ well-being. Finally, previous studies in Spanish workers suggest that, although non-work emotional social support can be an important predictor of mental health—especially during crises—, its impact on overall health is less consistent and context-dependent [77,78].

This study has the typical **limitations** of cross-sectional designs, which prevent establishing causal relationships between the variables analysed. Likewise, the use of non-probabilistic snowball sampling and voluntary participation through a self-administered online survey may have introduced self-selection bias, particularly among individuals more interested in the topic. Although the sample is representative of the university community at the University of Zaragoza, the results are not directly generalizable to other institutions or the entire Spanish university population due to the study’s contextual nature. Additionally, relying on self-reports means data are based on subjective perceptions, which may be influenced by memory bias or social desirability. Moreover, the large number of bivariate and subgroup tests increases the risk of chance findings; therefore, the results should be interpreted primarily in terms of effect sizes and confidence intervals, and the consistency of associations across models, rather than isolated p-values. Nevertheless, this study provides a valuable initial approach, contributing empirical knowledge on the psychosocial health of university staff and laying the groundwork for future research. It is advisable that future studies use random sampling to allow greater generalization and longitudinal designs to examine the evolution of these variables over time.

## Conclusion

This study has enabled an in-depth analysis of various psychosocial and occupational variables among university staff, identifying key factors influencing their overall health. Among these, perceived stress stands out as the most consistent predictor, followed by technostress, resilience, job satisfaction, workload, and social support. The combination and impact of these factors vary according to contract type, seniority, and job position, highlighting the need for differentiated approaches tailored to the characteristics of each group.

The findings offer important practical implications for the development of institutional strategies aimed at promoting the health of university personnel. Identifying perceived stress, technostress, and low resilience as key health determinants provides a solid foundation for designing future preventive interventions focused on strengthening personal and professional competencies.
